# Therapeutic use of calpeptin in COVID-19 infection

**DOI:** 10.1042/CS20220638

**Published:** 2022-10-21

**Authors:** Jameel Inal, Ainura Paizuldaeva, Esmeralda Terziu

**Affiliations:** 1 School of Human Sciences, London Metropolitan University, U.K.; 2 Biosciences Research Group, School of Life and Medical Sciences, University of Hertfordshire, U.K.

**Keywords:** calpeptin, COVID-19, Extracellular Vesicles, therapy

## Abstract

This perspective considers the benefits of the potential future use of the cell permeant calpain inhibitor, calpeptin, as a drug to treat severe acute respiratory syndrome coronavirus 2 (SARS-CoV-2) infection. Recent work has reported calpeptin’s capacity to inhibit entry of the virus into cells. Elsewhere, several drugs, including calpeptin, were found to be able to inhibit extracellular vesicle (EV) biogenesis. Unsurprisingly, because of similarities between viral and EV release mechanisms, calpeptin has also been shown to inhibit viral egress. This approach, identifying calpeptin, through large-scale screening studies as a candidate drug to treat COVID-19, however, has not considered the longer term likely benefits of calpain inhibition, post-COVID-19. This perspective will reflect on the capacity of calpeptin for treating long COVID by inhibiting the overproduction of neutrophil extracellular traps potentially damaging lung cells and promoting clotting, together with limiting associated chronic inflammation, tissue damage and pulmonary fibrosis. It will also reflect on the tolerated and detrimental *in vivo* side-effects of calpain inhibition from various preclinical studies.

## Introduction

Severe acute respiratory syndrome coronavirus 2 (SARS-CoV-2), on entering the upper respiratory tract and infecting type II alveolar cells, causes severe inflammation [1,2]). This hyperinflammation (so-called cytokine storm or cytokine release syndrome) from recruited immune cells forms a cycle of chronic inflammation, ultimately damaging lung tissue [3]. As the angiotensin-converting enzyme 2 (ACE2) receptor is widely expressed in many organs, infection of the gastrointestinal, renal and cardiovascular systems is also common alongside acute systemic inflammatory symptoms [4]. As a blood pressure regulator in the lungs, ACE2 controls the renin–angiotensin system. By balancing the activity of ACE, ACE2 offers protection of the lungs from acute injury, but this is disturbed upon the viral spike protein (S protein) binding ACE2, leading to acute injury with associated chronic inflammation and resultant lung fibrosis [5]. Despite the development of vaccines against COVID-19, because of the delay in vaccinating the world’s population, people are still getting infected and becoming seriously ill. There is therefore an ongoing need to develop drugs able to inhibit SARS-CoV-2 infection but also with antifibrotic and anti-inflammatory capacity [6].

This perspective will discuss the future use of calpeptin, the cell permeant cathepsin/calpain inhibitor, as a possible anti-SARS-CoV-2 drug. It will focus on calpeptin’s capacity to inhibit: (i) viral entry and (ii) extracellular vesicle (EV) release and viral egress. However, these reports have not commented on additional benefits of calpain inhibition, especially important in post-COVID-19. This article will therefore also reflect on calpeptin’s inhibition of (iii) neutrophil extracellular trap (NET) formation [7] and (iv) inflammation [8], tissue damage and pulmonary fibrosis (PF) [9].

## Calpeptin as an inhibitor of SARS-CoV-2 uptake

### Inhibition of SARS-CoV-2 entry

SARS-CoV-2 has two possible entry mechanisms, thus broadening its tissue tropism: (i) in transmembrane serine protease 2^+^ (TMPRSS2^+^) cells, a rapid entry is achieved in a pH-independent manner, cells being activated rapidly at the cell surface. (ii) In cells lacking or with low-level expression of TMPRSS2, the virus is endocytosed and sorted to endolysosomes where activation is pH-dependent.

**Figure 1:**
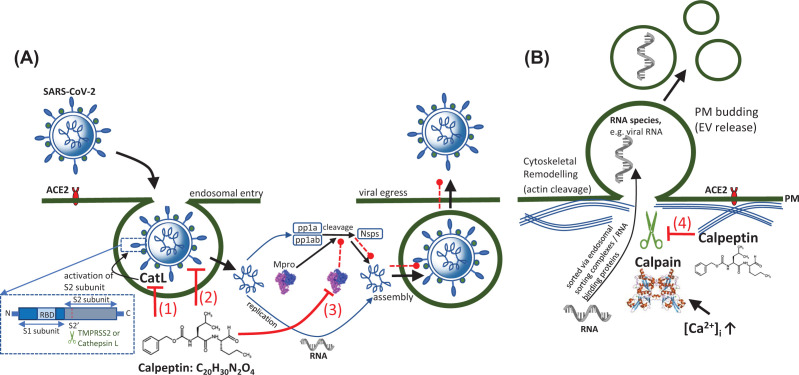
Calpeptin-mediated inhibition of calpain and its effect on SARS-CoV-2 entry and egress (**A**) During SARS-CoV-2 endosomal entry, in TMPRSS- or low expressing cells, SARS-CoV-2 follows a slow, pH-dependent pathway. Here, calpeptin inhibits cathepsin L- (CatL-) mediated activation of the S2 subunit of the S protein, thereby blocking viral entry (1). Calpeptin can also block viral entry by high-affinity binding to the S protein RBD thereby blocking S protein: ACE2 interaction (2). Calpeptin binds with high affinity to Mpro thereby preventing cleavage of polyproteins pp1a and pp1ab into the nonstructural proteins 1–16, resulting in inhibition of assembly and viral egress (3). In (**B**), calpeptin inhibits calpain-mediated remodeling of the actin cytoskeleton, thereby inhibiting the release of shedding EVs which may incorporate various viral macromolecules (4). Any such regulation of EV release may help reduce EV-mediated fibroblast proliferation and pulmonary fibrosis.

Both pathways require activation of the S protein. After the receptor binding domain (RBD) within the S1 subunit has bound ACE2 on target cells, the conformationally altered S2 subunit mediates membrane fusion following proteolytic cleavage away of S1, at the S1/S2 boundary (S2′ site) (Figure 1A(1)). If following the rapid, pH-independent pathway, TMPRSS2 performs this cleavage and activation of the viral S protein. However, in TMPRSS2^−^ cells (Figure 1A(1)), the slower acid-activated route is followed utilizing the host protease, cathepsin L (CatL), found in acidic endo/lysosomal compartments. Where SARS-CoV-2 enters TMPRSS2^−^ cells through endocytosis, numerous studies have shown that CatL inhibitors can inhibit viral entry [10–12], thus pointing to the use of CatL inhibitor, calpeptin (Figure 1A(1)).

In work in which libraries were screened for small molecule inhibitors for repurposing as entry inhibitor drugs, calpeptin was identified as having activity in *in vitro* infectivity assays. In Vero E6 cells with either low or high ACE2 expression, calpeptin (‘SR-914’) showed an EC_50_ of 174 and 163 nM, respectively [13]. This study suggested several mechanisms of action, including blocking entry by preventing ACE2:S protein RBD interaction through high affinity binding of calpeptin to S protein RBD on the S1 subunit (Figure 1A(2)).

### Inhibition of SARS-CoV-2 main proteases

In a study to screen, using X-ray crystallography, 5000 approved drugs or those in clinical trials, that bind to SARS-CoV-2 Main protease, Mpro, also known as 3C-like protease 3CL^pro^, calpeptin was found to be the most potent of these post-entry inhibitors. It bound in the active site, demonstrating high antiviral activity (EC_50_ = 72 nM) [14]. Using a SARS-CoV-2 pseudotyped particles (PP) entry assay to evaluate binding, and entry inhibitors, calpeptin was identified as a potent entry inhibitor [15], thus also confirming previous studies [16,17]. The latter study used the calpain/cathepsin B inhibitor, MDL 28710.

The structures of M^pro^ complexed with calpain inhibitors II and XII, recently solved [18], revealed binding sites that support the empirically observed inhibition of the protease activity of SARS-CoV-2 M^pro^ [19]. This potentially reveals a strategy for inhibiting both M^pro^ (Figure 1A(3)) and CatL [18]. These inhibitors have a broader spectrum of activity, also demonstrating antiviral activities against other coronaviruses, including MERS-CoV [20].

## Calpeptin-mediated inhibition of extracellular vesicle/SARS-CoV-2-mediated release from infected cells

### Extracellular vesicle biogenesis

EVs are membrane-bound intercellular communicative vesicles [21]. Carrying receptor proteins, cytokines, miRNA, mRNA, bioactive lipids and various metabolites, they are released from a wide range of cells and found in all body fluids and interstitial spaces [22]. Classified according to their mechanism of biogenesis, EVs comprise exosomes, microvesicles (or microparticles/ectosomes, MVs) and apoptotic bodies (ApoBs). Exosomes (50–100 nm) have an endosomal origin, resulting from the intraluminal budding of early endosomes to generate multivesicular bodies (MVB) containing intraluminal vesicles, released as exosomes upon fusion of these MVBs with the PM. MVs (50 nm to 1 µm) are released by budding and fission of the PM. Membrane curvature is initiated by ceramide, generated from sphingomyelin by sphingomyelinase; MV release is also accompanied by a breakdown in the asymmetry of the lipid bilayer and exposition of phosphatidylserine on the outer leaflet. During apoptosis, and rearrangement of the cytoskeleton, ApoBs (1–5 μm) are released. EVs in this article will refer to MVs and exosomes.

### Targeting extracellular vesicle biogenesis pathways as a means of limiting viral infection

In infectious diseases, EVs play a plethora of roles in enhancing infection and immune evasion [23]. For some time, it has been known that EVs and viruses share elements of their biogenesis pathways [24,25]. EVs released from virally infected cells, besides carrying molecules from their parent cells, also harbor viral genetic elements and proteins [24] and may be considered as defective viruses. In studies of the β-coronavirus family, using the prototypic mouse hepatitis virus, as well as SARS-CoV-2, these viruses egress infected cells by lysosomal exocytosis [26], having been trafficked to lysosomes from Golgi apparatus and *trans*-Golgi network via late endosomes/MVBs. As both EV and virus biogenesis may occur at the PM or within endosomes using endosomal sorting complexes required for transport (ESCRT) machinery to complete membrane fission, this justifies the aim of inhibiting EV biogenesis from virally infected cells as a means of limiting infection.

EVs play significant roles in disease pathology. For example, procoagulant endothelial EVs are released due to endothelial damage, TNF-α [27], or complement activation [28] resulting in coagulation and venous thromboembolism, presented in COVID-19, as deep vein thrombosis or pulmonary embolism. Pharmacological regulation of EV release has already been investigated [29,30] and in the task of finding drugs able to limit viral infection, this is an obvious direction, as recently demonstrated [31]. Kongsomros et al. identified calpeptin to be the most effective EV inhibitor drug against SARS-CoV-2. As a Ca^2+^-activated neutral cysteine protease, calpain, once activated, binds cytoskeletal proteins which leads to not only deformation of the PM, promoting EV release, but also cell migration, cellular proliferation and apoptosis [32]. The inhibition of calpain suppresses the release of EVs [30,31,33,34] (Figure 1B). Showing dose-dependent inhibition of infectious SARS-CoV-2 particles (IC_50_ 0.6 μM in Vero-E6 cells), in combination with antivirals, specifically remdesivir, calpeptin had increased effectivity [31]. Previously, calpeptin was demonstrated to inhibit SARS-CoV replication *in vitro* (EC_50_ 2 μM; IC_50_ 17 μM) [35]. A plethora of EV inhibitory drugs have been identified, targeting cytoskeletal organization, endocytosis and lipid-related mechanisms. Therefore, such combination therapies may pose an interesting strategy, with the proviso that as the pathways involved in EV biogenesis share certain molecular components, off-target effects of such EV inhibitors are also considered.

## Calpeptin inhibition of NETs as a therapeutic target in pulmonary fibrosis

**Figure 2:**
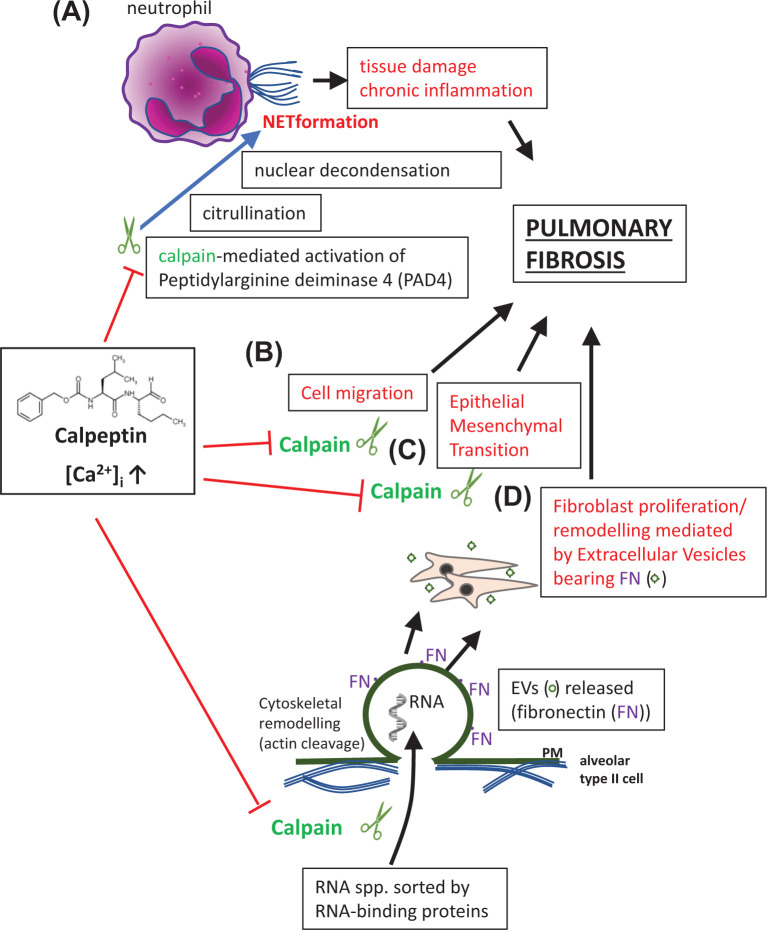
Calpeptin-mediated inhibition of calpain reduces inflammation and PF in COVID-19 (**A**) Calpleptin inhibits calpain activation of PAD4 and in turn citrullination, nuclear decondensation and NETosis-mediated tissue damage, inflammation and lung fibrosis. (**B**) Calpeptin inhibits calpain-mediated inflammatory cell migration and (**C**) EMT-mediated lung fibrosis, both leading to lung fibrosis. In (**D**), calpeptin inhibits calpain-mediated plasma membrane budding and fibroblast remodeling due to the FN bearing EVs.

The excessive release of NETs, webs of DNA extruded from neutrophils, containing enzymes able to sequester pathogens, is associated with tissue damage, chronic inflammation and has been implicated in PF [36]. Indeed, using an *in vitro* alveolar model, NETosis-induced epithelial–mesenchymal transition (EMT) following SARS-CoV-2 infection was deemed an important step leading to PF [37]. NETs therefore probably play a major role in COVID-19 pathology [38]. Peptidyl arginine deiminase 4 (PAD4) is up-regulated in COVID-19 in the lung and is essential in NETosis [39,40]. A pathway for NET formation was recently proposed that may be relevant for developing new COVID-19 therapies. This proposed that PAD4-mediated citrullination which induces nuclear decondensation requires calpain-mediated activation of the PAD4 enzyme (Figure 2A). In turn this synergizes with the calpain-mediated proteolysis of nuclear lamina and chromatin-bound proteins in the nucleus [41]. As a result, both PAD4 and calpain inhibition diminished the calcium ionophore-mediated, nuclear decondensation in neutrophils. This points to a further possible benefit of calpeptin in ameliorating PF, post-COVID-19.

## Calpain inhibition to reduce chronic inflammation and subsequent pulmonary fibrosis

According to current data, approximately 42% of COVID-19 patients develop acute respiratory distress syndrome (ARDS) [42]. As in the earlier SARS and MERS epidemics, ARDS in the COVID-19 pandemic was deemed a risk factor for fibrosis, but with added risk factors including old age and admission to intensive care. Even after removal of SARS-CoV-2, PF may continue to develop [43]. The proinflammatory state of ARDS, likely to be exacerbated in the elderly, is mediated by endothelial and epithelial injury from uncontrolled release of matrix metalloproteinases [44] and fibroproliferation. Together with proinflammatory cytokines TGF-β, VEGF, IL-6 and TNF-α, this may lead to PF in COVID-19 [45]. Fibrotic damage to lung tissue as occurs in PF is followed by release of a spectrum of cytokines identical to that described for COVID-19. The risk factors shared by both conditions include being male, elderly and having comorbidities such as diabetes and hypertension. The likely similar pathology of the lung disease could thus guide effective repurposing of drugs to treat severe COVID-19 [46].

### Calpeptin inhibition of cell migration and pulmonary fibrosis

Fibrosis occurs following a persistent insult to the lung or dysregulation of any of the four steps leading to wound healing [47]. Any of these stages therefore represent potential targets for antifibrotic therapy. Looking at lung inflammation, for some time we have known that calpain inhibitors show anti-inflammatory properties [48,49]. In COVID-19, the timing of any anti-inflammatory intervention, such as with corticosteroids or IL-1/IL-6 inhibitors is critical. Considering the three stages of COVID-19 proposed by Siddiqi and Mehra [50], anti-inflammatory therapies would be detrimental to administer during stage I (early infection) with high viral loads. It may be more appropriate, however, during the second stage of pulmonary involvement without hypoxia (IIa) and through phase IIb (by the end of which viral invasion has reached its minimum) through to the hyperinflammatory phase (stage III). Besides viral-mediated injury, bystander pathology of cells may be due to the influx of inflammatory neutrophils and monocytes. Calpeptin, as a calpain inhibitor can block integrin-mediated cell detachment [51]. It could therefore further block the infiltration of inflammatory cells (Figure 2B). Of note, calpain inhibition modulated cell migration (in tumor metastasis) by decreasing retraction of the rear of the cell by stabilizing linkages between integrins and the cytoskeleton [52].

### Calpeptin-mediated inhibition of EMT in pulmonary fibrosis

Following infection (or injury) to epithelial cells and subsequent inflammation and cell migration, fiboblast proliferation and differentiation into myofibroblasts by EMT is another potential target for antifibrotic therapy. An important recent investigation into potential therapies for TGF-β-induced fibrosis found that whilst translation of calpain 9 (CAPN9) induced by TGF-β caused myofibroblast differentiation in wild-type mice, *Capn9*^−/−^ mice, lacking CAPN9, were protected from fibrosis induced in heart, liver and lung [53]. Calpains, as cysteine proteinases that mediate Ca^2+^-dependent proteolysis of E-cadherin, are important contributors to organ fibrosis. In a mouse model of bleomycin (BLM)-induced PF, calpeptin inhibited IL-6, angiopoietin-1 and TGF-β1 production and fibrosis (attributed to collagen deposition) [46]. In other work, inhibition of calpain activity and ERK1/2 signaling in mice, reduced BLM-induced PF, supposedly through inhibition of EMT [54] (Figure 2C). This followed similar work where calpeptin treatment of BLM-induced PF in mice had been found to be antifibrotic through reduced EMT and TGF-β1-Smad2/3 signaling [55].

### Extracellular vesicle release in severe COVID-19 as a contributory factor in pulmonary fibrosis

A more recently considered factor contributing to fibrosis is that of EVs through disruptions in wound healing. Indeed, a recently described contributor to the pathology of PF was WNT-5a-mediated signaling via EVs, which stimulated fibroblast proliferation [56]. Furthermore, fibronectin (FN) expressed on the surface of these EVs, stimulates, at least *in vitro*, integrin α_5_β_1_ signaling and pathological fibroblast remodeling (Figure 2D). This is manifest as invasion and activation [17]. Part of the increased level of EVs, which is characteristic of severe COVID-19 and associated ARDS, is due to endothelial injury, whether released from the pulmonary capillary vasculature (angiotensin-converting enzyme^+^ [ACE^+^] [57]; von Willebrand Factor^−^ [vWF^−^] [58]) or systemic vasculature (ACE^−^; vWF^+^). EVs play a crucial role in the pathogenesis of PF. *In vivo* work has shown EVs release from injured endothelial cells to help develop PF [59]. This has also been supported by EV release from proinflammatory M2 alveolar macrophages [60]. Targeted pharmacological inhibition of EV biogenesis, as referred to above, may thus contribute to the growing arsenal of therapeutic interventions against COVID-19.

## Possible side-effects of calpeptin therapy, from *in vivo* studies

Calpains promote inflammation by a number of mechanisms leading to NF-κB activation and production of proinflammatory cytokines. They also result in recruitment of inflammatory cells and migration (as evidenced by calpain inhibition blocking integrin-mediated detachment of cells [51]). Furthermore, calpains increase leukocyte–endothelium interaction and thus plasma extravasation and diapedesis of inflammatory cells and this chronic inflammatory response eventually promotes fibrotic lesions. As mentioned earlier, there is considerable evidence in support of calpain inhibition as a means of protecting against tissue damage due to chronic inflammation. However, besides other positive effects, there is also evidence from preclinical studies of various detrimental effects [61].

By way of example of tolerated side-effects of calpeptin therapy, inhibition of calpain in preclinical models revealed itself to be neuroprotective following cancer chemotherapy [62], with no long-term detrimental side-effects. Furthermore, in mice, calpain-1 and -2 deficiency due to a tissue-specific or ubiquitous gene knockdown of *CAPNS1* was tolerated.

In terms of detrimental side-effects, *CAPNS1* knockout in aged mice and calpain-1 and -2 deficiency in muscle, resulted in dystrophy [63]. In mouse knockouts, muscular dystrophy was also caused by inhibition of calpain-3 [64]. Calpain-1 deficiency in mice and humans (due to *CAPN1* mutation) helped bring about ataxia [65]. In other work, *CAPN1* knockdown in mice affected platelet aggregation but with no adverse effect on bleeding times [66].

## Conclusions and perspectives

This article has described the inhibition of EV biogenesis as a way of limiting viral cell-to-cell transmission. Depending on the EV biogenesis pathway being targeted, there may be added benefits, especially in ameliorating PF in COVID-19, as mentioned above. Treating COVID-19 with calpain inhibitors such as calpeptin, a potent inhibitor or EV release [29,30] will provide not only antiviral activity but also potentially attenuate NET formation, inhibit EMT [55], chronic inflammation and PF. Although the focus has been on calpeptin, other EV inhibitors such as GW4869, which inhibits nSMase- (neutral sphingomyelinase-) mediated deformation of the PM (and was effective in limiting Zika viral infection [67]), may also be considered. This is because GW4869 can also reduce TNF-α release from macrophages [68] important in post-COVID-19 where TNF-α is a key inflammatory cytokine in associated ARDS and PF [69].

This perspective has summarized four significant roles of calpain inhibition in COVID-19, using the peptidomimetic calpain inhibitor, calpeptin. As a prospective drug, calpeptin has low toxicity having been tolerated in mice for up to 4 weeks [70]. Although calpeptin is not currently in clinical trials as a treatment for COVID-19, BLD-2660, a synthetic, small molecule inhibitor against calpain 1, 2 and 9 is in Phase 2 clinical trials to reduce IL-6 levels and attenuate fibrotic damage [71]. Moreover, calpeptin or other calpain inhibitors have been in clinical trials for a host of other conditions [72] or will be, having shown recent efficacy in preclinical studies [73]. Another potential therapy for post-COVID-19 PF is the use of mesenchymal stem cell-derived EVs [74]. However, since much effort has been put into finding new, isoform-specific calpain inhibitors [75], with many of these also in clinical trials [13,73], drug repurposing of such selective inhibitors seems particularly advantageous and should be pursued to treat not just acute COVID-19 but also to manage the long-term effects of post-COVID-19.

## Data Availability

Data sharing is not applicable to this paper as it is a perspective article and there are no data.

## Abbreviations

ACE: angiotensin-converting enzyme
ARDS: acute respiratory distress syndrome
BLM: bleomycin
CAPN9: calpain 9
EVs: extracellular vesicles
FN: fibronectin
MVB: multivesicular bodies
NET: neutrophil extracellular trap
NF-κβ: nuclear factor-kappa B
PAD4: peptidyl arginine deiminase 4
PF: pulmonary fibrosis
RBD: receptor binding domain
SARS-CoV-2: severe acute respiratory syndrome coronavirus 2
TNF-α: Tumour Necrosis Factor-alpha
